# Morphology of blood microbiota in healthy individuals assessed by light and electron microscopy

**DOI:** 10.3389/fcimb.2022.1091341

**Published:** 2023-01-18

**Authors:** Borislava Tsafarova, Yordan Hodzhev, Georgi Yordanov, Vladimir Tolchkov, Reni Kalfin, Stefan Panaiotov

**Affiliations:** ^1^ Department of Microbiology, National Center of Infectious and Parasitic Diseases, Sofia, Bulgaria; ^2^ Faculty of Chemistry and Pharmacy, Sofia University, Sofia, Bulgaria; ^3^ Institute of Neurobiology, Bulgarian Academy of Sciences, Sofia, Bulgaria; ^4^ Department of Health Care, South-West University “Neofit Rilski”, Blagoevgrad, Bulgaria

**Keywords:** blood microbiota, TEM, SEM, microscopy, morphology, proliferation cycle

## Abstract

**Introduction:**

The blood microbiome is still an enigma. The existence of blood microbiota in clinically healthy individuals was proven during the last 50 years. Indirect evidence from radiometric analysis suggested the existence of living microbial forms in erythrocytes. Recently targeted nucleic acid sequencing demonstrated rich microbial biodiversity in the blood of clinically healthy individuals. The morphology and proliferation cycle of blood microbiota in peripheral blood mononuclear cells (PBMC) isolated from freshly drawn and cultured whole blood are obscure.

**Methods:**

To study the life cycle of blood microbiota we focused on light, and electron microscopy analysis. Peripheral blood mononuclear cells isolated from freshly drawn blood and stress-cultured lysed whole blood at 43°C in presence of vitamin K from healthy individuals were studied.

**Results:**

Here, we demonstrated that free circulating microbiota in the PMBC fraction possess a well-defined cell wall and proliferate by budding or through a mechanism similar to the extrusion of progeny bodies. By contrast, stress-cultured lysed whole blood microbiota proliferated as cell-wall deficient microbiota by forming electron-dense or electron-transparent bodies. The electron-dense bodies proliferated by fission or produce in chains Gram-negatively stained progeny cells or enlarged and burst to release progeny cells of 180 – 200 nm size. On the other hand, electron-transparent bodies enlarged and emitted progeny cells through the membrane. A novel proliferation mechanism of blood microbiota called by us “a cell within a cell” was observed. It combines proliferation of progeny cells within a progeny cell which is growing within the “mother” cell.

**Discussion:**

The rich biodiversity of eukaryotic and prokaryotic microbiota identified in blood by next-generation sequencing technologies and our microscopy results suggest different proliferation mechanisms in whole and cultured blood. Our documented evidence and conclusions provide a more comprehensive view of the existence of normal blood microbiota in healthy individuals.

## Introduction

The blood of healthy individuals has traditionally been considered to be sterile. The detection of microbes in the blood is interpreted as an indication of infection. Reports of microbiota in whole blood associated with a variety of chronic diseases or transient and latent infections have appeared in the literature for many years. Nevertheless, the blood microbiome is still an enigma, and evidence for the existence of blood microbiota in healthy individuals is steadily accumulating.

The term blood microbiome refers to the collection of genomes or genomic fragments from all the microorganisms, that is the DNA and/or RNA, while microbiota refers to viruses (Liang and Bushman, 2021; Cebriá-Mendoza et al., 2021), bacteria (Damgaard et al., 2015) and fungi (Panaiotov et al., 2018) found in the blood. Next-generation sequencing technology has been used to identify viruses, bacteria, archaea, and fungi in the blood of healthy individuals (Morgan and Huttenhower, 2012; Paisse et al., 2016; Panaiotov et al., 2021). Nevertheless, the distinction between whether those genetic materials belonged to viable or dead microorganisms or microbial cell-free nucleic acids is controversial. Convincing supportive evidence for the presence of microbiota in the blood of healthy individuals has been a few. (Tedeschi et al., 1969; Domingue and Schlegel, 1977; Kalfin, 1997; Damgaard et al., 2015; Markova et al., 2015; Dimova et al., 2017; Markova, 2017; Panaiotov et al., 2018; Cebriá-Mendoza et al., 2021). For several decades, the scientific community debates whether normal microbial flora could be present in the blood of healthy individuals. Some researchers suggest that healthy blood microbiota may be transient circulatory microorganisms (Goraya et al., 2022). The disputes are related to the evidence for the existence of blood microbiome and blood microbiota. Researchers are divided into three groups. The most recent group, probably founded with the publication of G. Tedeschi in 1969, supports the hypothesis, that blood is not as sterile as previously supposed (Tedeschi et al., 1969). Blood microbiota are naturally existing in the blood from birth throughout the whole life of the individual as dormant, latent, or non-culturable microbial forms (Markova et al., 2016; Dimova et al., 2017). The researchers supporting the hypothesis for the existence of blood microbiota claim that the normal blood microbiota are not harmful to the host and should be considered commensal microbes, living in harmony within the host’s blood ecosystem. Experimental evidence that such a balanced state could exist between microbes and host is the mass immunization with live bacterial or viral vaccines. Live attenuated vaccines are derived from laboratory-weakened (attenuated) bacteria and viruses, so that when injected they will persist, infect cells and replicate causing no or only very mild disease - i.e. BCG immunization and reimmunization of children and adults. The pediatric dose for primary immunization of infants at birth contains approximately 0,025 mg moist weight of BCG and between 0.75–3.0 ×10^5^ viable units. BCG vaccination is widely applied to billions of children worldwide and practiced since 1921. BCG vaccination in infancy and adolescence induces immunological memory of mycobacterial antigens that are still present and measurable for at least 14 years in the majority of vaccinated individuals (Weir et al., 2008). Other authors claim that in mice viable BCG bacilli persisted for at least 16 months post-vaccination (Kaveh et al., 2014). At the same time, the blood cultures of BCG-vaccinated individuals are negative. The logical question is where do these live BCG cells disappear? It could be supposed that the BCG bacteria persist in the host as non-culturable cell-wall deficient forms (Markova et al., 2015). Many viruses can persist in the blood and tissues in lytic or latent forms. Viral persistence may be maintained for a lifetime. Persistent human papillomavirus infection lies not within its ability to cause infection but in the ability of the virus to establish long-term persistence in the host (Della Fera et al., 2021). Measles viral RNA persisted in the blood, respiratory tract, or lymph nodes four to five times longer than the infectious virus and the clearance of MeV RNA from blood is prolonged (Lin et al., 2012). For many other microbial species, this could be an option to parasitize in the blood.

The other hypothesis assumes that identified microbial DNAs in the blood are cell-free circulating DNAs (Gosiewski et al., 2017; Kowarsky et al., 2017; Szilagyi et al., 2020; Zozaya-Valdés et al., 2021) or vesicles of microbial origin containing microbial metabolites and fragmented DNAs or RNAs (Chronopoulos and Kalluri, 2020; Ricci et al., 2020). The idea of a blood microbiome is controversially accepted. The presence of nucleic acids or microbiota in the blood of healthy individuals is considered transitory and mainly attributed to pathology associated with microbial translocations from the gut, mouth, and skin injuries (Emery et al., 2021). The third group denies the existence of enigmatic blood microbiota in healthy individuals (Mitchell et al., 2016; Martel et al., 2017; Zozaya-Valdés et al., 2021; Tan et al., 2022). Convincing confirmation of blood microbiota will emerge by applying sophisticated techniques, including microscopy.

The existence of blood microbiota in clinically healthy individuals was proven over the last 50 years (Castillo et al., 2019). The microbial content in the blood of healthy animals was also studied. A significant number of bacterial species were detected in the blood of control groups of healthy chickens (Mandal et al., 2016) and cats by NGS analysis (Vientos-Plotts et al., 2017). Based on the fact that an increasing number of studies identify the presence of foreign microorganisms in healthy human blood and that blood microbiota does not necessarily equate with infection or with a diseased state (Tedeschi et al., 1969; McLaughlin et al., 2002) we accept that these microbial structures could be assumed as normal resident blood parasitizing microorganisms. In our previous study, we identified blood microbiota in 100% of the blood samples, and a rich microbial biodiversity by targeted sequencing of 16S rDNA and internal transcribed spacer (ITS) markers of cultured and non-cultured blood samples (Panaiotov et al., 2021). We tested the capacity of a specific medium to resuscitate and culture the blood microbiota of healthy individuals. Our results demonstrated that the majority of blood microbiota were culturable, thus their proliferation cycle could be studied.

Many bacteria can be visualized by electron microscopy in clinical samples but most of them cannot be grown in culture. We are limited to culturing all bacteria, but in nature non-culturable do not exist. They all multiply and spread. Cryptic microorganisms, whether intra- or extracellular, are ubiquitous. Special and appropriate conditions are needed to be disclosed. Few research groups reported light and electron microscopy evidence regarding the morphology of persisting blood microbiota in diseased individuals (Domingue and Schlegel, 1977; Markova, 2020), but still, survival and life cycle mechanisms in a host are not clear. It was assumed that blood microbiota may exist in healthy individuals but it is difficult to predict how they proliferate and are controlled by the host. The existence of viable and proliferating microbial structures in the blood of healthy individuals is still under discussion. In our previous studies, we identified that blood microbiota under stress activate survival mechanisms and started to proliferate. We identified that high concentrations of vitamin K and cultivation at 43°C induced blood microbiota proliferation. In the present study, we describe light and electron microscopy observations of proliferation mechanisms of blood microbiota in peripheral blood mononuclear cells isolated from freshly drawn blood and cultured lysed blood. We selected to study the human peripheral blood mononuclear cells because they are critical components of the immune system and are involved in both humoral and cell-mediated immunity. If blood microbiota exist, it would be logical to look for them in the PMBC fraction. Lysed and 0.22 µm filtered blood is appropriate for studying cryptic intracellular microorganisms.

## Materials and methods

Bioethics committee approval of the study (decision 38/14.07.2016) by the Institute of Neurobiology, Bulgarian Academy of Sciences (BAS, Sofia, Bulgaria) and individual written consent were obtained.

### Healthy volunteers

The healthy volunteers were selected according to the following criteria: age between 20 and 60 years old; weigh at least 50 kg; not be pregnant; and not suffer from severe fatigue, insulin-dependent diabetes, or infectious diseases; no hospitalizations or blood transfusions during the last two years; no medical treatments with antibiotics or systemic corticoids, dental treatment, surgical intervention, body piercing during the last six months. The 7 healthy individals were 3 women and 4 men aged from 24 to 58 years old.

### Samples

Three ml of blood was collected from seven clinically healthy adults in Vacutainer tubes with heparin and K_3_EDTA as anticoagulants (Vacutainer K3E and Vacutainer Heparin Tube, BD, USA). All samples were previously characterized for their microbial biodiversity content by applying 16S rDNA and ITS targeted next-generation sequencing (Panaiotov et al., 2021). Two hundred µl of all blood samples were tested for sterility by growing on Sabouraud and blood agar 90 mm plates at 37°C for 72 hours.

### Culture

For microscopy analysis we cultivated and isolatied blood microbiota in stress culture by a modified resuscitation strategy previously developed by Emil Kalfin (Kalfin, 1997). Blood was lysed in 3 volumes of sterile water for 1 hour at room temperature. In order to remove unlysed cells and membranes the blood lysate was filtered through 0.22 µm membrane filters (Sartorius, Gottingen, Germany). Two hundred µl of lysed and filtered blood were added to 1.8 ml of culture medium. Culturing was performed in sterile 2 ml polypropylene tubes. The culture base medium was composed by Brain Hearth Infusion (BD, NJ, USA) medium and 0.2% yeastolate (BD, NJ, USA) adjusted at pH 6.8 and sterilized. Sterile (D+) sucrose at 10% final concentration and water-soluble form of vitamine K_3_ - menadione sodium bisulfite (Sigma-Aldrich, USA) at 1 mg/ml were sterilized by filtration, then added to the base medium. Resuscitation growth was induced at 43°C. Culturing was performed for 7 hours and 14 tubes were collected at 30 min intervals. Aliquots were set aside for light and electron microscopy experiments. For TEM an aliquot was fixed by adding 1:1 (v/v) 4% glutaraldehide.

### Light microscopy

Microscope slides were soaked in 2% hydrochloric acid alcohol (100 ml of 95% ethanol and 2 ml of concentrated 37% hydrochloric acid) for two hours, and then rinsed thoroughly with sterile distilled water. Microscope slides of PMBC and cultured lysed blood were Gram stained, and observed under immersion at magnification 1000×.

### Dark-field microscopy

Observation by dark-field microscopy was performed with an immersion dark-field condenser (Pancratic) of an optical microscope Amplival (Carl Zeiss, Jena, Germany), equipped with a photo-tube adapter and 24 MP camera (Canon 800D, Canon Inc., Tokyo, Japan). We used ethanol cleaned microscope slides of 1.0 mm thickness and cover glasses of 0.17 mm thickness. Ten µl of cultured or non-cultured blood sample was applied to the microscope slide and covered with a cover glass.

### Scanning electron microscopy

Scanning electron microscopy (SEM) was performed by using SEM LYRA I XMU (Tescan Ltd., Czech Republic). Lysed blood cultured samples were fixed for 1 h at room temperature in phosphate-buffered (PBS) 2.5% glutaraldehyde (Merck, Germany). Ten microliters were placed on microscope glass slides, dried and dehydratated in 50%, 70%, 85%, 95% and 100% ethanol in increasing concentration for 5 min each. Since these specimens are non-conductive and can collect electrostatic charge from the electron beam (resulting in image artifacts), the samples were coated with carbon to be made electrically conductive and were electrically grounded to prevent accumulation of electric charge. Coating of materials was performed with a Quorum K450X carbon sputtering system (Quorum Ltd., UK). SEM observation was performed at accelerating voltage of 10 kV by detection of secondary electrons (SE). Secondary electrons are formed from the conduction or valence bands of the specimen by inelastic scattering interactions with the electron beam. These electrons are then detected by an Everhart–Thornley detector and the signal is transformed into an image. Samples were observed at different magnifications ranging from 10,000 to 120,000x.

### Transmission electron microscopy

Whole blood samples for TEM observations were processed within 1 hour after collection. Sterile heparinized blood collected in a Vacutainer tube (BD, USA) was diluted 1: 3 with PBS pH 7.4 (Thermo Fisher Scientific, USA) and centrifuged at 400 g for 30 min on Histopaque-1077 (Sigma-Aldrich, USA) density gradient. The peripheral blood mononuclear cells were removed from the gradient, washed 3 times in PBS, then washed in RPMI 1640 (Sigma-Aldrich, USA) medium with 10% FCS (Sigma-Aldrich, USA) and processed for TEM. The cells were resuspended in 3% low-gelling temperature agarose (Sigma-Aldrich, USA) and put on ice. The solidified agarose was cut into 1 mm^3^ cubes, and then fixed in 2.5% glutaraldehyde (Sigma-Aldrich USA) and postfixed in 1% osmium tetraoxide (Sigma-Aldrich, USA) and dehydrated in increasing concentrations of 30, 50, 70, 96% of ethanol for 10 min each, 100% ethanol for 2 × 20 min and propylene oxide for 2 × 20 min. Specimens were impregnated in propylene oxide:Durcupan ACM (Sigma-Aldrich, USA) 1:1 and embedded in Durcupan ACM. Polymerisation was carried out at 60°C for 18 h in an oven. The samples were thin cut to 20 30 nm and observed under a High-Resolution Transmission Electron Microscope (HRTEM) JEOL JEM 2100 model at an accelerating voltage of 200 kV.

For TEM analysis of cultured blood samples the following methodology was applied: Blood samples were cultured in resuscitation media for blood microbiota. Culturing was performed for 7 hours and 14 tubes were collected at 30 min intervals. Tubes were centrifuged at 12.000 rpm for 5 min. The pellet was fixed overnight at 4°C with 4% glutaraldehyde in 0.1 M sodium cacodylate buffer, pH 7.4 (Sigma-Aldrich, USA), and then centrifuged. Collected pellets were mixed with 3% agarose. Agarose cubes were then postfixed in 1% osmium tetraoxide (Sigma-Aldrich, USA) in 0,1 M cacodylate buffer and dehydrated in increasing concentrations of 30, 50, 70, and 95% of ethanol for 15 min each, 100% ethanol for 2 × 20 min and propylene oxide for 2 × 20 min. Specimens were impregnated in 2:1 propylene oxide:Durcupan ACM (Sigma-Aldrich, USA), propylene oxide:Durcupan ACM 1:1 and propylene oxide:Durcupan ACM 1:2 for 30 min each and embedded in Durcupan ACM. The polymerisation in gelatin capsules was carried out at 56°C for 48 h. The samples were ultra-thin cut to 20-30 nm sections, contrasted with uranyl acetate and observed under a High-Resolution Transmission Electron Microscope (HRTEM) JEOL JEM 2100 model at an accelerating voltage of 200 kV.

## Results

The concept of the existence of normal blood microbiota in healthy individuals was challenged with light and electron microscopy techniques. To describe the morphology of the observed cell types we adopted the terms proposed by Domingue (Domingue and Schlegel, 1977; Domingue, 1995), “mother” cell corresponds to a mature cell giving origin to progeny cells and an “electron-dense body” a mother cell that is electron dense.

### Light microscopy of freshly drawn blood

Slides with peripheral blood mononuclear cells isolated from freshly drawn blood or lysed whole blood samples were Gram-stained and tested for bacteria on light microscopy at 1000× magnification with an oil immersion objective. The analysis was negative.

### Transmission electron microscopy

**Figure 1:**
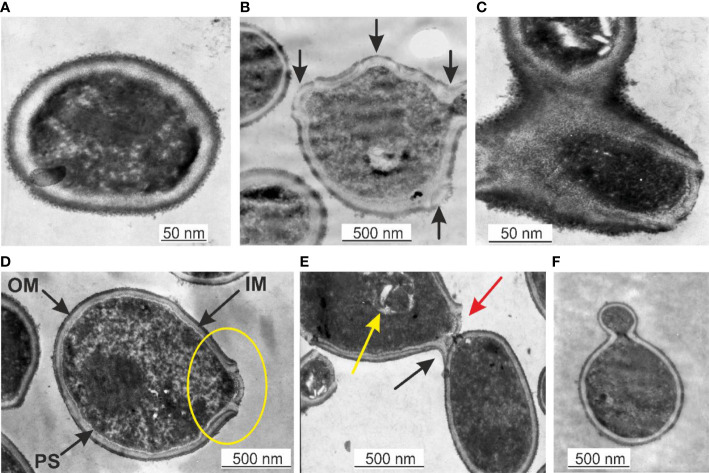
**(A)** Primary cell with a mossy cell wall. Bar 50 nm. **(B)** Cell surface protrusions where new progeny cells erupt (black arrows). **(C)** Junction of progeny cells (septum). **(D)** Ultrastructures of blood microbiota. Black arrows indicate the inner membrane (IM), intermediate translucent periplasmic space (PS), outer membrane (OM), and progeny extrusion site (yellow ring). **(E)** A progeny cell is attached to the “mother” cell (black arrow), a progeny cell prepared for extrusion (red arrow), and a progeny cell inside the “mother” cell (yellow arrow). **(F)** Classical budding.

TEM is a suitable technique to study the morphology of specific microbial structures at high resolution. TEM was applied to study the proliferation mechanisms of blood microbiota among the peripheral blood mononuclear cells isolated from freshly drawn blood and in cultured blood samples. In fresh blood samples, processed within one hour after collection, we observed cell-walled microbial structures that proliferate by extrusion of progeny cells from many sides sprouting on the microbial surface. The “primary cell” or “primary body” appeared spheroidal of about 180–200 nm that increases in size. The surface of the growing cell appeared mossy (**Figure 1A**). A cell wall was evident, distinguishing a well-defined inner membrane (IM), translucent periplasmic space (PS), and outer membrane (OM). The cytoplasm appeared with light and opaque sections. The lighter structures appear to be the bacterial nuclei. The growing primary cell could give birth to progeny cells. The progeny cell starts to form inside the enlarged primary cell. The cell wall opens and a progeny cell is extruded (**Figure 1B, D**). The cell wall opening mechanism starts with the appearance of a bulge on the cell surface, followed by a spherule opening through which a progeny cell is extruded, like a “bleb from a mouth” (**Figure 1D**, yellow ring). For a short time, the progeny cell could remain attached to the “mother vesicle” (**Figure 1C, E**). The progeny cells could be extruded in different places on the “mother’s cell” surface (**Figure 1B**). We observed at least four sites where progeny cell extrusion could have occurred (**Figure 1B**). A chain of connected progeny cells can form, but we did not observed long chains of cells (**Figure 1C, E**). We also suspect another mechanism of cell division in which the “mother” cell reproduces by septum formation and subsequent septum division into daughter cells (**Figure 1C**). Categorical confirmation of such a mechanism of cell division has not been observed. Classical microbial proliferation by budding was also observed (**Figure 1F**).

The observed by us cell wall morphology corresponded to Gram-negative bacteria composed of a thin peptidoglycan layer (~ 5 nm) squeezed between two distinct bilayer membranes, the cytoplasmic (inner) and the outer membrane. The mossy bacterial cellular envelope could be of lipopolysaccharide origin.

### Results from cultured blood samples

**Figure 2:**
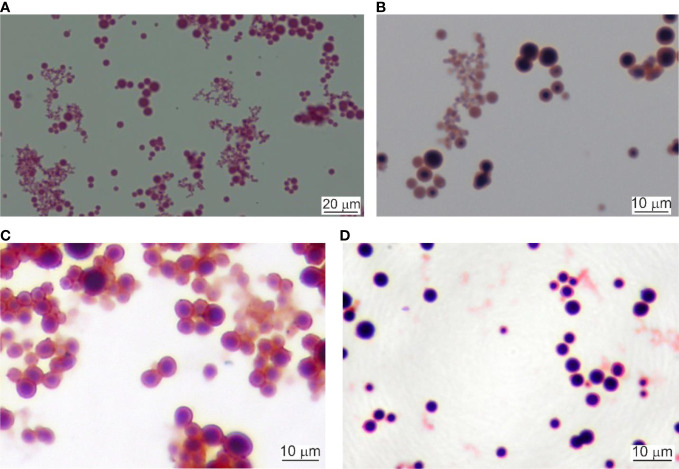
Light microscopy of cultured blood microbiota. **(A)** Gram staining at 4 hours of culturing, 800×. Scale bar 20 µm. **(B)** Gram staining at 6 hours, 1000×. **(C, D)** Cultures stained at 24 hours of culturing, 1000×. Scale bar 10 µm.

Lysed and 0.22 µm filtered whole blood samples were cultured as described. Initially, the cultures were light red. After the first hour of culturing the cultures started to turn cloudy. Tiny, Gram-stainable round particles appeared under 100× magnification. The color changed to light brown. After the second hour, the microscopic field was full of single cells, chains, and groups of round cells. At the third hour, the culture color was already brown. Between the fourth and seventh hour cultures became dark brown and visible sediment was formed. After centrifugation, the cell mass increased as measured by the number of cells per microscopic field. In our previous experiments (Panaiotov et al., 2018) we identified several morphological forms of Gram-stained blood microbiota observed at light microscopy: *i.* “dense bodies” of Gram positively stained microbial structures; *ii.* Gram positively stained bodies surrounded by Gram-negative coat and *iii.* “dense bodies” producing in chains of Gram negatively stained progeny cells (**Figure 2**). The “dense body” could divide by fission, or produce in chains Gram negatively stained “primary” cells. Light microscopy proved rapid proliferation of the blood microbiota.

### Dark-field microscopy

**Figure 3:**
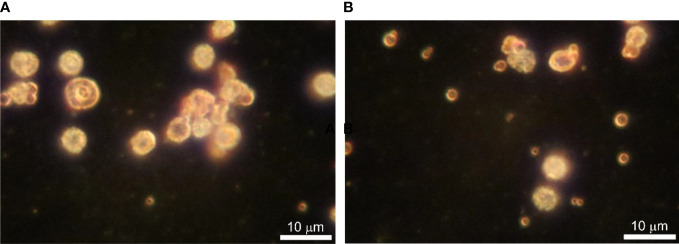
**(A, B)** Dark-field microscopy of *in vitro* cultured lysed blood in BHI broth supplemented with vitamin K at 43 ^○^C for 24 h. Bar 10 µm.

Dark-field microscopy proved to be an appropriate method to observe fresh samples of blood microbiota at greater contrast without the need for fixation, staining, and drying. Cells are seen as white spots on a dark background. Cells move by Brownian movement. Considering the simplicity of the technique it is easily applied. The images obtained were of high quality and informative. We observed three types of objects in preparations from cultures of lysed and filtered blood samples: *i.* very small cells, as glowing dots in size under 500 nm that can only be seen by their Brownian motion at a higher light intensity, *ii.* bigger spheroidal structures about a micron or two with a shell and a central “core”, and *iii.* 4–5 µm cells that have a grainy internal structure and rough edges (**Figure 3**). The size of the observed by us structures rules out the possibility that these could be of viral origin. With appropriate continuous imaging equipment, the live cell proliferation could be observed.

### Scanning electron microscopy of cultured blood samples

**Figure 4:**
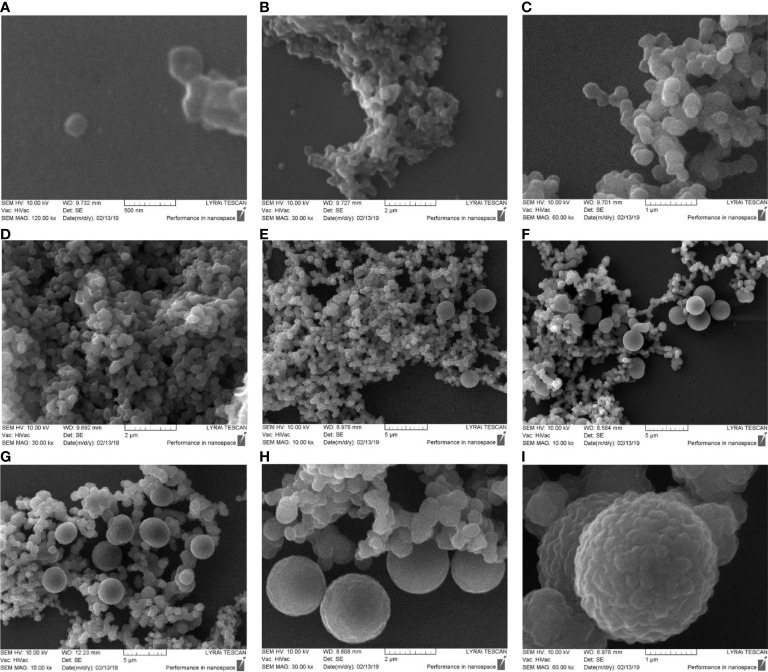
SEM images of cultured blood microbiota. Blood samples were lysed, 0.22 µm filtered and cultured for 7 hours. **(A, B)** SEM analysis of cultured blood for 1 hour. **(C–E)** cultures at 2 – 4 hours. **(F–H)** cultures at 5 – 7 hours. **(I)** SEM morphology of big “mother” cell after 7 hours of culturing.

Lysed and 0.22 µm filtered blood samples were cultured for 7 hours. Starting after the first hour of cultivation we set aside at 30 min intervals aliquots for analysis. Samples were centrifuged at 12.000 rpm and the pellet was processed for scanning electron microscopy. The first sample before cultivation was negative for blood microbiota. The pellet was composed of blood debris. Next sample was set aside after 1 hour of cultivation. SEM analysis visualized single cell-like structures of 200 nm size in correlation with light microscopy and TEM (**Figure 4A**). We observed rapid proliferation of cell mass. The pellet was composed of tiny or groups of Gram-stained particles/cells also visible under light microscope which increased in numbers with cultivation time (**Figures 4B, C**). Between the first and fourth hours of cultivation the preparations analysed be SEM demonstrated little variations (**Figures 4D, E**). After 5 hours and 30 min of cultivation we observed morphologically heterogeneous microbial forms and appearance of big spheroidal cells (>2 nm) with rough surface among groups of cells of smaller size (**Figure 4F**). From the big cells extruded in chains progeny cells. Proliferation by budding could be also observed. After 7 hours of cultivation the big cells divided by simple fission or proliferated by budding (**Figures 4G, H**). At 60 000 magnification the big “mother” cells appeared with rough surface (**Figure 4I**), also observed by dark-field microscopy and TEM. The surface roughness is characterized by protrusions which give birth to offspring of elementary cells in chains or by budding. It could be supposed that the “mother” cell is a sack of progeny cells. TEM analysis gave support for such a conclusion (**Figure 6**, **7**).

### Transmission electron microscopy of cultured blood samples

**Figure 5:**
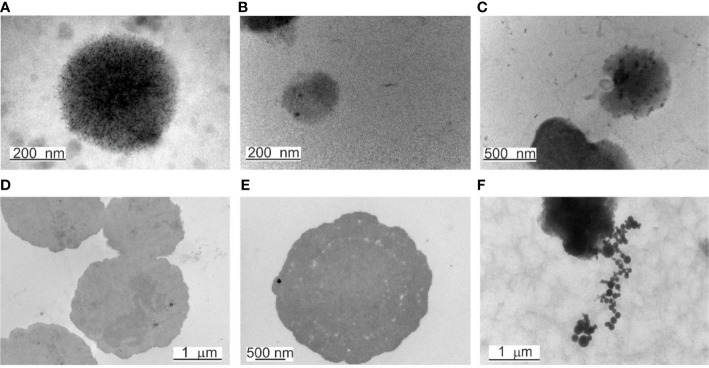
TEM of elementary bodies and electron-dense bodies. **(A)** A mossy elementary body. **(B)** Elementary body of 180 – 200 nm in size. **(C)** Extrusion of an elementary body. **(D, E)** Large electron-dense bodies, named also “mother” cells. **(F)** In chains proliferation of electron-dense bodies.

**Figure 6:**
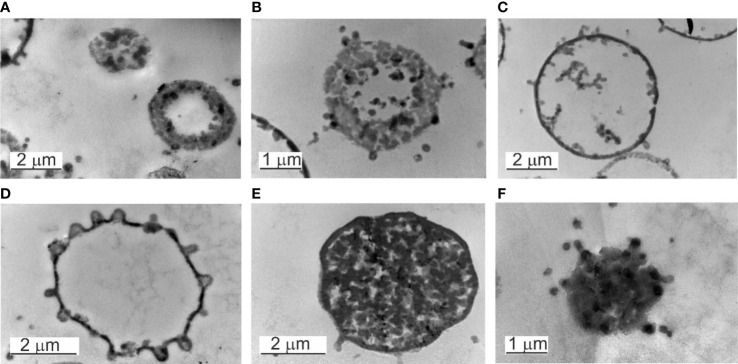
Electron transparent and electron-dense bodies. **(A–D)** Electron transpatent bodies. **(B)** The burst of an electron-transparent body. **(C, D)** The elementary bodies growing within the “mother cell” can leave at any site of the cell surface. **(E)** Electron-dense body in a growth phase. The cytosol is full of progeny cells. **(F)** The burst of an electron-dense body.

Fine-structure studies by TEM analysis distinguished two types of mature “mother” cells. Their morphology is the opposite. We observed electron-dense large “mother” cells that appeared as black spots (**Figure 5D, E**) and electron-transparent “mother” cells delimited by a membrane (**Figure 6A–D**). The primary structure is an elementary mossy cell of 180 – 200 nm size (**Figure 5A, B**). A series of observations demonstrated that the electron-dense “mother” cell enlarged and proliferated by budding or extrusion of progeny cells in chains (**Figure 5F**). We also observed the disintegration of the dense “mother” body into elementary cells. The elementary cells multiplied rapidly, enlarged to giant “mother” cell structures in size >2 µm, and end their proliferation cycle by releasing numerous new elementary cells visible as small granules after the burst of the giant “mother” cell structures. **Figure 5C** shows rarely observed extrusion of an elementary body by indentation of the membrane.

Another series of observations demonstrated that the “mother” cell enlarged and disintegrated into elementary cells (**Figure 6E**). The elementary cells multiplied rapidly, enlarged to giant “mother” cell structures in size >2 µm, and end their proliferation cycle by releasing numerous new elementary cells visible as small granules after the burst of the “mother” cell (**Figure 6F**).

Inside the electron-transparent “mother cells” we observed on the membrane surface budding edges from which progeny cells were expelled. Before that, the progeny cells appeared to adhere to the inner side of the “mother`s” membrane. Once passed the membrane by budding and released into the surrounding fluid medium, further growth occurred repeating the proliferation cycle.

**Figure 7:**
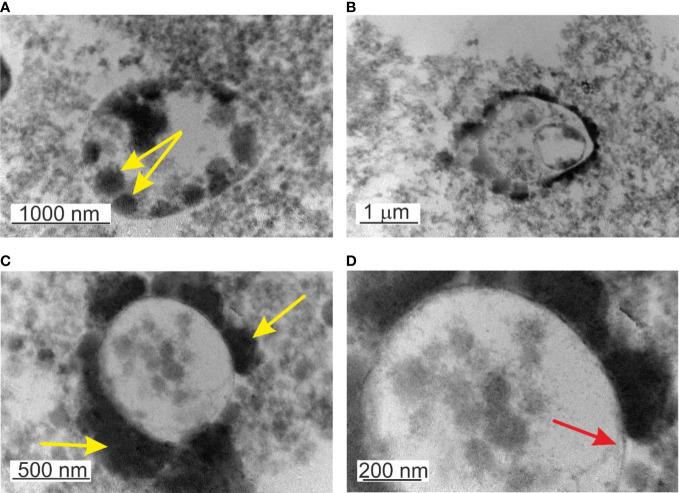
TEM images of lysed, non-filtered, and cultured blood. **(A)** Mature cell >2µm filled in with elementary cells - yellow arrow. **(B)** Growth of progeny cells within a progeny cell that grows within the “mother” cell **(C)** Formation of thick capsular material on the external side of the membrane or attached elementary cells. **(D)** Microbial-like structures delimited by a membrane – red arrow.

TEM observations of non-filtered blood demonstrated particular features. The majority of the observed “mother” cells were electron transparent. During maturation, the “mother” cell transforms into a reproductive sack. The growing “mother” cells were filled with progeny cells (**Figure 7**). Two types of “mother” cells were observed: *i*. “mother” cells only delimited by a membrane (**Figure 7A**) and *ii.* cells with a membrane covered by a thick extracellular matrix (**Figure 7B, C**). The matrix could be of peptidoglycan origin. The cell envelope resembled gram-positive bacteria.

Another particular feature was the observation of a structure “cell within a cell”, a growing transparent progeny cell delimited by a membrane within the “mother” cell (**Figure 7B**). Interestingly the inner progeny cell was also filled by progeny cells adhered to the inner side of the membrane. This particular rare case of blood microbiota proliferation combines the growth of progeny cells within a progeny cell that is growing within the “mother” cell. All structures mature and proliferate in common. To our knowledge, this is an example of a new matryoshka-like mechanism of microbial proliferation that previously has not been described for blood microbiota.

## Discussion

Cryptic bacteria have been isolated or indirectly confirmed in patients with chronic or latent infections including nephritis (Ponig et al., 1972), rheumatic fever (Carapetis et al., 2016), idiopathic hematuria (Domingue et al., 1993), Crohn’s disease (Rathnaiah et al., 2017), mycobacterial infections and many others (Domingue and Woody, 1997). Next-generation sequencing and ultramicroscopic studies have identified an authentic blood microbiome in several non-communicable diseases (Potgieter et al., 2015) and healthy individuals (Paisse et al., 2016; Panaiotov et al., 2018; Castillo et al., 2019).

Till recently life sciences experts considered the blood to be an immunologically well-controlled and sterile environment, and that invasion of microbes into the blood will lead to a transitory infection or infectious disease. This perception changed over the past 50 years by studies reporting the presence of blood microbiota and circulating microbial metabolites in the blood of humans and animals (Mandal et al., 2016; Vientos-Plotts et al., 2017; Castillo et al., 2019; Goraya et al., 2022). Nevertheless, the blood microbiome is still an enigma evidence for the existence of blood microbiota in healthy individuals is steadily accumulating. In our experiments with blood collected from healthy individuals, we could not relate the observed microbial forms in peripheral blood mononuclear cells isolated from freshly drawn blood to invasion of the host (sepsis) or pathology. We consider that these microbial structures naturally persist in the host blood from early birth. Moreover, we consider that the blood of healthy individuals has its microbiota.

The direct and indirect evidence for the existence of viruses, bacteria and fungi in the blood of healthy individuals, residing as normal microflora has thrown light on their impact on host immune surveillance, and possibly on their role in chronic systemic inflammatory diseases (Potgieter et al., 2015; Cebriá-Mendoza et al., 2021). Models describing the mechanisms for bacterial translocation from the gastrointestinal tract to extraintestinal sites, such as the extracellular interstitial space (“tissue space”) of the body and bloodstream, predict pathology associated with: (a) disruption of the ecologic gastrointestinal equilibrium allowing intestinal bacterial overgrowth, (b) increased permeability of the intestinal mucosal barrier, and (c) deficiencies in host immune (Schatten et al., 1955; Berg, 1999). On the analogy of the gastrointestinal tract microbiota, which live in symbiosis with the host, it is evident that translocated gut microbiota to blood form a well-controlled and balanced symbiosis with the host. The coexistence logic is associated with the depletion of microbial antigenicity and the main benefit for the host could be the maturation, modulation, and maintenance of the immune system. This could be compared to lifelong vaccination with a live vaccine (i.e. BCG Halling-Brown et al., 2008). The underlying mechanisms of this strict control are not known. We suppose that a microbial translocation is a natural event, not obligatory associated with pathology in the host. The origin of the normal population of microbiota in blood associated with microbial translocations through the intestinal barrier, oral cavity, or skin surface has yet to be confirmed. In this sense, it is not necessary to have intestinal epithelial barrier damage for microbial translocation. Intestinal permeability for microbial translocations might be a necessary natural process for the internal organs and systems to communicate with the external microbial world. Blood microbiota are in natural symbiosis with the host similar to the symbiosis between the microbiota and the skin, mucous membranes, gut, and others, thus coexisting with human cells, causing no classical pathology.

Paisse et al. in 2016, demonstrated that a diversified microbiome exists in healthy blood. Most of the blood bacterial DNA was found located in the buffy coat cell blood fraction (93.74%), while erythrocytes contain more bacterial DNA (6.23%) than the plasma (0.03%) (Paisse et al., 2016). Studies reported associations between blood microbiota and chronic diseases (Domingue and Woody, 1997; Potgieter et al., 2015; Visser et al., 2019; Hammad et al., 2020). The current opinion about the origin of blood microbiota is associated with microbial translocation, an event known also as atopobiosis, through the gut (Gautreaux et al., 1994; Berg, 1999), oral cavity (Koren et al., 2011; Amar and Engelke, 2015; Seringec et al., 2015; Emery et al., 2021) or skin (Visser et al., 2019). The majority of the microbial translocations occur through the gut forming the so-called axis, i.e. gut-brain axis, heart-gut axis, gut-liver axis, etc. Pathologies associated with microbial translocations involving mesenteric lymph nodes (Gautreaux et al., 1994), liver (Pinzone et al., 2012), spleen, or urogenital system have been reported (Domingue et al, 1993). A significant number of studies visualized cell-wall deficient microbial structures or L-forms found in diseased individuals (Fernandes and Panos, 1977; Errington et al., 2016). Recent studies demonstrated that the internal organs and tissues of healthy and diseased individuals possess their microbiomes (Salihoglu and Önal-Süzek, 2021). Associations of tissue dysbiosis with cancer, neurodegenerative, and inflammatory diseases have been reported (Pisa et al., 2015; Nejman et al., 2020; Poore et al., 2020). It has been found that breast cancer tissue has a specific microbial profile (Meng et al., 2018) and brain disorders such as Huntington’s disease and Alzheimer’s disease suggest that brain tissue microbial colonization is a risk factor (Alonso et al., 2019).

To confirm the presence of normal blood microbiota in healthy individuals we performed on peripheral blood mononuclear cells isolated from freshly drawn blood and on lysed and filtered cultured blood samples comparative analysis by combining light microscopy, dark-field microscopy, scanning electron microscopy, and transmission electron microscopy observations. These and our previous results (Panaiotov et al., 2018; Panaiotov et al., 2021) confirmed the presence of blood microbiota in clinically healthy individuals composed of microbial structures demonstrating particular life cycle features. We identified different modes of proliferation of blood microbiota in peripheral blood mononuclear cells isolated from freshly drawn blood and in cultured lysed blood. The cultured blood microbiota in liquid medium demonstrate classical growth dynamics of a microbial culture with lag, log and plateau and death or dormant phases (data not shown).

In peripheral blood mononuclear cells isolated from freshly drawn blood, we observed microbial spheroidal cells with a cell wall composed of inner and outer membranes. Blood microbiota species were controlled by a few mechanisms of proliferation. The growth of progeny cells inside the “mother” cell and extrusion of the progeny cell from the “mother” body was the predominant mode of proliferation. Budding was also observed (**Figure 1**). Extrusion of progeny cells in chains was suspected but not confirmed. It could be expected that the resident species in blood persist as few morphological forms.

Cultured blood samples demonstrated microbiota delimited by a membrane that resemble cell-wall deficient L-form bacteria. The life cycle of the blood microbiota in culture turns out in several steps. First, spheroidal microbial L-form cells originating from lysed blood, give origin to offspring that developed within the cytoplasm of the growing vegetative “mother” cell. The cell membrane could be covered by a thick extracellular matrix (**Figure 7A**). During maturation, the “mother” cell transforms into a reproductive sack. We observed two types of “mother” cells, electron-dense bodies (**Figures 5D** and **6E**) and electron-transparent bodies (**Figure 6C**). Progeny cells could pass the “mother” cell membrane at any site (**Figure 6D**). The progeny cells increased in numbers and burst the cell membrane (**Figure 6B, F**). We concluded that the “dense body” is composed of “primary” cells that grow in the cell cytosol and then leave or burst the body membrane. It seems that there is a transition state between transparent and electron-dense bodies. Probably different species of blood microbiota adopt a specific form of development forming transparent or electron-dense bodies. We observed semi-electron dense bodies that accumulate and liberate progeny bodies after burst (**Figure 6E, F**). Domingue (Domingue, 1995; Domingue and Woody, 1997) and Markova (Markova, 2017; Markova, 2020) described electron-dense bodies and cytoplasmic particles in chronic infections and in healthy individuals which resemble our observations for blood microbiota. Blood microbiota in culture behaves as stable L-forms that could not revert to the original bacteria.

Our results correlate and confirm previous findings (Kalfin, 1997; Domingue, 1995; Domingue and Woody, 1997; Markova, 2017; Markova, 2020);. The described light and electron microscopy microbial structures and proliferation cycle mechanisms were not dissimilar to those previously described for cell wall-deficient, L-form bacteria (Green et al., 1974; Domingue and Schlegel, 1977; Domingue, 1995; Domingue and Woody, 1997; Leaver et al., 2009; Errington, 2017). In contrast to classical bacteria, L-forms can reproduce by a great variety of unusual modes, such as irregular binary fission, budding, tubulation, vesiculation, protrusion-extrusion of elementary bodies and granules from large bodies, multiple division with intracellular fragmentation of cytoplasm or combination of all types (Prozorovskiï et al., 1981; Errington, 2017). Part of these reproductive models was observed by us in blood microbiota.

Applying standard procedures for DNA staining with acridine orange, DAPI (4′,6-diamidino-2-phenylindole), and Hoechst 33342 for fluorescence microscopy we observed a high level of autofluorescence in preparations of cultured blood microbiota. Staining results were negative and most probably were due to an unsuitable staining procedure linked to cell permeability. By TEM we observed a thick shell covering the cell bodies (**Figure 7C**). We did not test other staining conditions. It is supposed that the life cycle of blood microbiota encompass intracellular proliferation mainly in leukocytes and erythrocytes. Documented evidence by Pohlod et al. (1972) indicated the presence of cell-wall deficient microbial forms in circulating erythrocytes by fluorochrome staining. The authors examined freshly drawn blood and described structures, which appear to be microbial, extended in rhizoid filaments from the erythrocytes (Pohlod et al., 1972).

We found that by cultivation the blood microbiota membrane was covered by a thick cell coat, capsule, or shell (**Figure 7C, D**). The fact that it took us a lot of experimental trials to develop a reliable method for DNA extraction from blood microbiota, which was continuously improved for cultured and non-cultured blood samples, suggested that the thick coat might protect the cells from stress. These particular features of the cell coat might be associated with survival mechanisms such as dormancy, aggregation, and shielding leading to immune neutrality, i.e. a complete lack of cellular and humoral immune responsiveness.

We observed that slight variations in the culturing conditions could affect the staining capacity, morphology, size, and shape of the blood microbiota. Similar changes associated with the changing environmental conditions were also observed by other authors (Markova, 2020). Our results suggest that blood microbiota adopted few mechanisms to proliferate and can assume a form quite different from that of a free-living microorganism.

This study has some limitations linked to the microscopy morphological analysis that impacted and influenced the interpretation of the findings. By light or scanning microscopy we could not precisely the microbial origin of the observed structures – bacteria or fungi? We suspect that bacteria dominate. Fungi were not ruled out but characteristic fungal features were not observed or not definitive, i.e. dark-field observations. Another weakness of our study is that viruses were not targeted. We focused on observations of bacteria and fungi. The size of the “elementary cells” is less than 200 nm. These “enigmatic” life microbiota structures pass through 0.22 µm filter membrane pores. The study demonstrated microbiota cell wall and membrane structures. The intracellular life cycle of the blood microbiota was not studied. Our future studies will aim at their intracellular persistence and growth. In our experimental cultures, the persisting microbiota in the blood after subculturing cannot revert to culturable bacteria. This limits the possibility of fully studying their physiology.

The rich biodiversity of eukaryotic and prokaryotic microbiota identified in the blood of healthy individuals identified by NGS analysis requires more deep analysis of the different mechanisms of proliferation in peripheral blood mononuclear cells isolated from freshly drawn and cultured blood. Future experiments will elucidate the mechanisms of intracellular survival of blood microbiota in erythrocytes and other blood cells.

## Conclusions

TEM images demonstrated well-defined cell structures of native blood microbiota (**Figure 1**). TEM and culture results confirmed that the blood microbiome represents viable structures rather than debris resulting from the degradation of blood elements, such as lipids or hemoglobin complexes. We observed that blood microbiota undergo complex life cycles in peripheral blood mononuclear cells isolated from freshly drawn and cultured blood, involving different morphological transformations. Like L-forms, blood microbiota can reproduce by a variety of modes, such as irregular binary fission, budding, protrusion-extrusion of progeny cells from large electron-dense bodies, vesiculation, tubulation, enlargement of sack with elementary progeny cells and burst or combination of all types. We also observed a new matryoshka-like proliferation phenomena called by us “a cell within a cell” (**Figure 7B**). Our documented evidence and conclusions provide a more comprehensive view of the existence of normal blood microbiota in healthy individuals. Future studies should elucidate the intracellular mechanisms of the proliferation of blood microbiota in blood cells in healthy individuals.

## Data availability statement

The raw data supporting the conclusions of this article will be made available by the authors, without undue reservation.

## Ethics statement

The studies involving human participants were reviewed and approved by Bioethics committee approval of the study (decision 38/14.07.2016) by the Institute of Neurobiology, Bulgarian Academy of Sciences (BAS, Sofia, Bulgaria) and individual written consent were obtained. The patients/participants provided their written informed consent to participate in this study.

## Author contributions

SP, GY and RK: Conceptualization, investigation, supervision, writing the original draft, and preparation. BT: Collection of data, reviewing, and editing. YH and VT: Reviewing and formatting the manuscript. SP and RK: Funding. All authors contributed to the article and approved the submitted version.
